# Structural and conformational rearrangements of human serum albumin, transferrin, and blood plasma induced by carbosilane dendrimer therapeutic delivery system

**DOI:** 10.1038/s41598-025-23355-7

**Published:** 2025-11-12

**Authors:** Serafin Zawadzki, Elżbieta Okła, Sylwia Michlewska, Tomasz Makowski, Adam Buczkowski, Paula O. López, Francisco J. de la Mata, Maksim Ionov, Maria Bryszewska, Katarzyna Miłowska

**Affiliations:** 1 Department of General Biophysics, Faculty of Biology and Environmental Protection, University of Lodz, 141/143 Pomorska St., Lodz, 90-236 Poland; 2 Bio-Med-Chem Doctoral School of the University of Lodz and Lodz Institutes of the Polish Academy of Sciences, University of Lodz, Matejki 21/23, Lodz, 90-237 Poland; 3 Laboratory of Microscopic Imaging and Specialized Biological Techniques, Faculty of Biology and Environmental Protection, University of Lodz, Banacha12/16, Lodz, 90-237 Poland; 4 Centre of Molecular and Macromolecular Studies, Polish Academy of Sciences, Sienkiewicza 112, Lodz, 90-363 Poland; 5 Division of Biophysical Chemistry, Department of Physical Chemistry, Faculty of Chemistry, University of Lodz, Pomorska 163/165, Lodz, 90-236 Poland; 6 Department of Organic and Inorganic Chemistry, IQAR, University of Alcalá, Madrid, 28805 Spain; 7 Networking Research Center on Bioengineering, Biomaterials and Nanomedicine (CIBER-BBN), Madrid, 28029 Spain; 8 Ramón y Cajal Health Research Institute (IRYCIS), Madrid, 28034 Spain; 9 Faculty of Medicine, Collegium Medicum, Mazovian Academy in Plock, 2 Dabrowskiego Sq, Plock, 09-402 Poland

**Keywords:** Protein corona, Carbosilane dendrimer, Plasma protein interactions, Hemocompatibility, Biochemistry, Biophysics, Biotechnology, Chemistry, Materials science, Nanoscience and technology

## Abstract

Upon intravenous administration, nanoparticles quickly become enveloped by plasma proteins, lipids, and sugars, forming a biomolecular corona that redefines their biological identity and directs systemic distribution. In this study, we examine a third-generation, positively charged, PEGylated carbosilane dendrimer engineered for the delivery of therapeutic siRNA across the blood-brain barrier. Employing a comprehensive array of analytical techniques—including fluorescence quenching assays, circular dichroism spectroscopy, isothermal titration calorimetry, atomic force microscopy, and transmission electron microscopy—we characterize the dendrimer’s binding kinetics and thermodynamics, and its impact on structure of human serum albumin, transferrin and complex environment of plasma. Our findings reveal that the dendrimer induces distinct conformational rearrangements at both tertiary and secondary levels, with thermodynamic analyses indicating that the interactions are predominantly driven by favorable entropy changes and multivalent binding. Furthermore, the dendrimer appears to promote a reorganization of plasma components, potentially leading to aggregation and misfolding reminiscent of fibril formation. Collectively, these findings provide critical insights into the more profound understanding of nanoparticle-protein interactions affecting the biodistribution and hemocompatibility of nanovectors and lay a foundation for further research aimed at safer and more effective therapeutic applications.

## Introduction

A wealth of work has been published providing extensive insight into the evaluation and potential applications of dendrimer-based nanosystems in treating brain diseases^1–8^. Our research investigated a third-generation, positively charged, PEGylated carbosilane dendrimer and its complex with siRNA targeted against the apolipoprotein E4 (APOE4) gene, a major genetic risk factor for Alzheimer’s disease^9^. Recognizing that the hemocompatibility of intravenously administered nanoparticles is critical to their therapeutic efficacy and safety, our study examined explicitly the dendrimer’s interactions within plasma. Accordingly, in this manuscript we focus exclusively on dendrimer–plasma interactions. Upon injection into the bloodstream, nanoparticles rapidly encounter plasma proteins, lipids, and sugars, which immediately cover their surface, forming a “biomolecular corona”. This process leads to the development of a new “biological identity” for the nanoparticles. The nanoparticle’s size, shape, and surface chemistry, which is collectively termed “synthetic identity”, directly influences the formation of a biomolecular layer on the nanoparticle surface. Both the nanoparticle’s synthetic identity and its acquired biological identity dictate the nanoparticle fate *in vitro* and, ultimately, in systemic responses^10,11^. The inherent complexity and variability of biomolecular layers conjugated with a nanovector arise from the diverse interactions and functions of macromolecules within the physiological environment. Proteins play a critical role in shaping the biomolecular corona. The Plasma Proteome Database has annotated a total of 7614 plasma protein products derived from 3778 unique genes, highlighting the plasma proteome as one of the most complex and diverse among body fluids^12^. Initially, proteins abundant in serum bind to the nanoparticle surface, forming a transient “soft corona” that is later replaced by less abundant but higher affinity proteins, resulting in the formation of the “hard corona”. This competitive adsorption phenomenon is known as the Vroman effect^13^. The formed protein corona is a highly dynamic 3D assembly of proteins that are bound to each other^14^. Comprehensive data analysis across multiple studies has identified a distinct set of proteins, termed the “adsorbome”, exhibiting a high affinity for nanomaterials. The analysis suggests that a typical plasma protein corona comprises approximately 2–6 high-abundance proteins and a larger pool of low-abundance proteins^15^. The adsorbed proteins’ composition and conformation can significantly influence the fate and impact nanoparticles’ biological functionality by either enhancing or hindering it. Nanoparticles can disrupt native protein conformation by inducing partial unfolding, misfolding, aberrant secondary structures, oxidative damage, or complete denaturation. These alterations not only trigger an immunogenic response but also compromise critical protein-protein interactions, cellular signaling, and DNA transcription processes^16^. Nanoparticles can induce conformational changes in proteins that lead to fibril formation associated with neurodegenerative diseases such as Parkinson’s, Alzheimer’s, and Creutzfeld-Jacob disease. Fibrillation occurs when partially or fully unfolded proteins aggregate into β-sheet-rich structures, which subsequently elongate and organize into large, insoluble amyloid fibrils. Linse et al. demonstrated that nanoparticles—including copolymers, cerium oxide particles, carbon nanotubes, and quantum dots—accelerated the fibrillation of β2-microglobulin by concentrating proteins on their surfaces, thereby facilitating oligomer formation^17^. In contrast, gold nanoparticles have been found to inhibit this fibrillation process, highlighting their potential as therapeutic agents for these conditions^18,19^. The altering of adsorbed proteins’ structural and functional properties may result in immune cells’ recognition and uptake. Specifically, such interactions could induce abnormal unfolding that leads to the formation of novel “cryptic” conformational epitopes or destabilize native protein structures to reveal previously hidden continuous epitopes^20^. Recognition by the immune system may also be caused by direct opsonization, such as the adsorption of immunoglobulins, coagulation proteins, and complement proteins. These reduce the circulation time of nanoparticles by enhancing recognition by the macrophage receptors and clearance by the mononuclear phagocyte system (MPS), consequently leading to inflammation, nanoparticle accumulation in organs, and even immune system impairment. Conversely, adsorbed dysopsonins, including most apolipoproteins, decrease nanoparticle clearance, extending their circulation time and prolonging their *in vivo* persistence, ultimately enhancing their efficacy.

Among the various plasma proteins typically found in the protein corona, apolipoproteins significantly influence the ability of the nanoparticles to cross biological barriers. Specifically, the presence of Apo B and Apo E has been shown to enhance the uptake of liposomes by hepatocytes, and Apo A-I, Apo E, and Apo B-100 significantly improve the transport of nanoparticles into the central nervous system^21–23^. The variation in systemic biodistribution arises from cell-type-specific receptor expression profiles, which may cause enhanced internalization depending on the protein corona composition. At the same time, the protein corona can hinder internalization by creating a protein layer between the nanoparticle and the cell membrane. This steric hindrance reduces the nanoparticle’s ability to interact with cell surface receptors and impedes internalization. The exact mechanism mitigates the nanovector’s cytotoxic effects on vessel walls and blood components, such as eryptosis^24,25^. Furthermore, the protein corona can modulate nanoparticle-thrombocyte interactions influencing thrombogenicity^26,27^.

To counteract these adverse effects—building on the success of attaching polyethylene glycol (PEG) chains to therapeutic proteins for improved systemic delivery— PEGylation of nanoparticles has become a widely adopted strategy for enhancing efficiency and safety. PEG is often favored due to its well-documented biocompatibility, long history of safe use in humans, and classification as Generally Regarded as Safe (GRAS) by the Food and Drug Administration (FDA). By forming a hydrated steric barrier around the nanoparticle, PEG effectively reduces protein adsorption, minimizes nanoparticle aggregation, and limits opsonization and phagocytosis, thereby enhancing therapeutic efficacy. Consequently, PEGylated nanovectors exhibit prolonged systemic circulation, reduced immunogenicity, and improved stability in various biological environments. PEGylation mitigates hemolysis and facilitates nanoparticle penetration through challenging biological barriers, such as the BBB. Although PEG reduces non-specific protein adsorption, studies have shown that PEGylated surfaces still acquire a protein corona. Notably, all these effects depend highly on PEG architecture factors such as chain length and surface density^28–31^.

A comprehensive understanding of nanocarrier–blood protein interactions is essential for optimizing nanoparticle-based delivery systems. Our objective was to develop a nanovector that evades immune recognition, prolongs systemic circulation, and efficiently transports siRNA across the blood-brain barrier, thereby offering a promising therapeutic strategy for Alzheimer’s disease^9^. This study assesses protein interactions with the third-generation carbosilane dendrimer G3Si PEG6000, specifically evaluating its interactions with human serum albumin, transferrin, and whole plasma. By elucidating the mechanistic aspects of protein corona formation, we establish a foundation for future assessments of its impact on blood morphotic elements, hemocompatibility, and therapeutic efficacy.

## Materials and methods

### Dendrimer

**Fig. 1:**
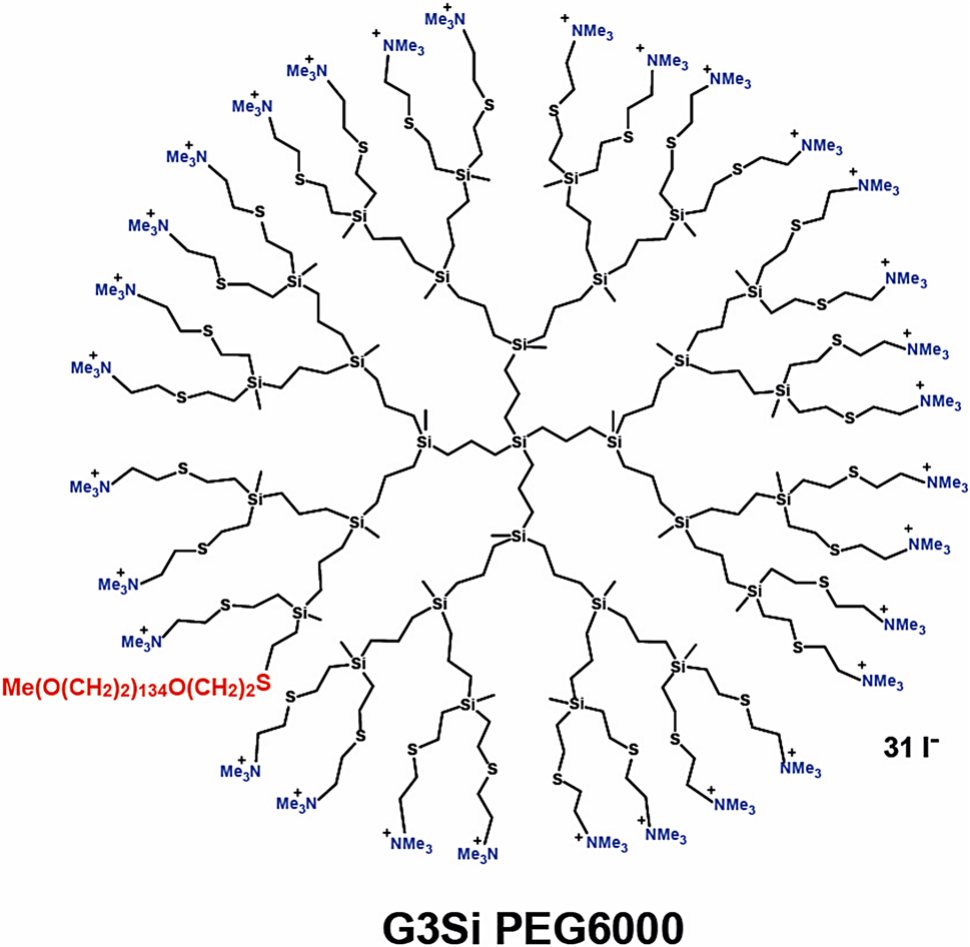
Structure of studied dendrimer G3Si PEG6000.

Dendrimer with a silicon atom core, surface tertiary ammonium groups, and single polyethylene glycol (PEG) (6000) – G3Si PEG6000 with chemical formula C_601_H_1324_I_30_N_31_O_135_S_32_Si_29_ and molar weight 16794.81 g/mol is presented in Fig. 1. The studied dendrimer was diluted in a phosphate buffer solution of 7.4 pH. The synthesis and biophysical characterization of the dendrimer, along with its complex with siRNA, was described in previous work^32^.

### Other reagents

Human serum albumin (HSA) was obtained from Sigma-Aldrich (St. Louis, MO, USA), while human transferrin (Tf) was sourced from Biorbyt Ltd. (Cambridge, UK). Additional chemical reagents, including sodium dihydrogen phosphate and disodium hydrogen phosphate, were sourced from Chempur (Piekary Śląskie, Poland). All reagents were of analytical grade, and solutions were prepared using Milli-Q purified water to ensure high purity and reproducibility.

### Fluorescence quenching of HSA and Tf

Tryptophan fluorescence quenching of HSA and Tf (1 µM) was studied by titrating increasing concentrations of dendrimer G3Si PEG6000 (4–20 µM). Measurements were conducted at 25 °C in 1-cm quartz cuvettes using a Perkin-Elmer LS-55 spectrofluorimeter (Perkin-Elmer, Waltham, MA, USA). The excitation and emission wavelengths were at 305 and 445 nm, with the excitation and emission slit widths being set to 2.5 and 9 nm, respectively. The experiment was performed in 3 repetitions.

### Circular dichroism of HSA and Tf

Circular dichroism (CD) measurements were performed to examine HSA and Tf (0.5 µM) in the presence of the stepwise increasing concentration (0.5–20 µM) of the G3Si PEG6000 dendrimer, without isolating complexes. The experiments were conducted using a Jasco J-815 CD spectrometer (Jasco International Co., Ltd., Tokyo, Japan) with 5-mm path length quartz cuvettes at a constant temperature of 25 °C. CD spectra of the proteins alone and in the presence of the dendrimer were measured between 195 and 260 nm. Thus, the resulting signals of the proteins in the presence of the dendrimer represents ensemble averages of free protein, free dendrimer, and complexed species. The percentage of the protein secondary structure content in the presence of the G3Si PEG6000 dendrimer was calculated using CDNN circular dichroism spectroscopy deconvolution software version 2.1 (Applied Photophysics Ltd., Surrey, United Kingdom). All samples were prepared in a 10 mM phosphate buffer, and the same buffer was used as the baseline for the measurements. The experiment was performed in 3 repetitions.

### Isothermal calorimetric titration of HSA

An isothermal calorimetric titration (ITC) (LV Affinity ITC with gold cells, TA Instruments) was performed in order to measure the thermodynamics of interactions between HSA and the G3Si PEG6000 carbosilane dendrimer in aqueous 10 mM phosphorous buffer solution pH 7.4. HSA solution (182 µl; 10 µM; in cell) was titrated by adding 31 of 5 µl doses of a 0.2 mM dendrimer solution (from the syringe). Pre-isolation of complexes was not used. The time interval between successive injections was 300 s. Measurements of the thermal effects of the titration were carried out at 25 °C with a stirring rate of 125 RPM in an auto-equilibrate mode. During the titration, the molar ratio of dendrimer to HSA, increased from 1.1/1 to 27/1. The thermal effects of diluting the dendrimer solution (in the syringe) in 10 mM phosphorous buffer solution pH 7.4 (in the cell) were determined independently, maintaining the same calorimeter parameters as for the proper titrations. The thermal effects of the direct interaction of HSA with dendrimer were calculated by subtracting the effects of dendrimer dilution from the corresponding thermal effects of the protein solution’s titration with the dendrimer solution. The determined binding isotherms describing the thermal effects of the direct interaction of HSA with studied dendrimers as a function of the titrated solution composition were analyzed in NanoAnalyze software by non-linear multiparameter regression using the independent-site model to calculate the stoichiometric parameter, equilibrium binding constant and the standard thermodynamic functions describing the process of receptor macromolecule (protein) binding with molecules of studied dendrimers: enthalpy, entropy and Gibbs free energy.

### Atomic force microscopy of HSA and plasma

The morphology of HSA, plasma, and their conjugates with the G3Si PEG6000 dendrimer was analyzed using atomic force microscopy (AFM) on droplets deposited onto mica (Grade V-1 Muscovite). Whole-blood donations from *n* = 3 healthy donors were collected by the Regional Centre for Blood Donation and Haemotherapy in Lodz into CPD (citrate–phosphate–dextrose) anticoagulant blood-bag systems. All experiments used individual-donor plasma (no pooling was applied). Human plasma was separated from blood by centrifuging it at 4200 rpm for 15 min. Conjugates were prepared by mixing HSA or plasma with the dendrimer in PBS and incubated at room temperature for 20 min. AFM measurements were conducted using a Flex Axiom Nanosurf apparatus equipped with a C3000 controller (Nanosurf AG, Liestal, Switzerland). The experiments employed dynamic force mode (tapping mode) and phase imaging to capture detailed surface characteristics. The analysis was performed using HI’RES-C14/CR‐AU µmasch probes with a typical tip radius of < 1 nm, a spring constant of 5 N/m, and a resonance frequency of 160 kHz. The resulting images were recorded at a resolution of 512 × 512 data points. Image processing and analysis were conducted using the Scanning Probe Image Processor (SPIP) software (Image Metrology, Hørsholm, Denmark).

### Transmission electron microscopy of HSA and plasma

The morphological characteristics and spatial distribution of HSA, plasma, and their conjugates with the G3Si PEG6000 dendrimer were analyzed using a JEOL-1010 transmission electron microscope (Japan) at 100,000× magnification. Whole-blood donations from *n* = 3 healthy donors were collected by the Regional Centre for Blood Donation and Haemotherapy in Lodz into CPD (citrate–phosphate–dextrose) anticoagulant blood-bag systems. All experiments used individual-donor plasma (no pooling was applied). Human plasma was separated from blood by centrifuging it at 4200 rpm for 15 min. The conjugates were formed by mixing HSA or plasma with the dendrimer in PBS at room temperature for 20 min. A molar ratio of 1:9 HSA to dendrimer was employed, representing the optimal saturation ratio determined through ITC analysis, with the dendrimer concentration set at 36 µM. For plasma samples, the same dendrimer concentration was added to a 55% plasma solution to prepare 100 µL solution. 15 µL of either the conjugate or the unbound protein was applied to a 200-mesh copper grid coated with carbon and allowed to adhere for 10 min. Both samples were stained with a 2% (w/v) uranyl acetate solution for 20 min to enhance contrast. To enhance legibility, images have been sharpened.

## Results and discussion

Despite extensive efforts to minimize the protein adsorption on nanoparticle surfaces, complete elimination of this phenomenon remains unattainable. The inevitable formation of a protein corona has profound implications for nanoparticle behavior, influencing their biological fate, stability, and therapeutic efficacy. This realization has redirected many research efforts toward understanding the relationship between nanocarrier surface properties and nanoparticle-protein interactions. We employed a comprehensive suite of analytical techniques, including fluorescence quenching, circular dichroism, isothermal titration calorimetry, transmission electron microscopy, and atomic force microscopy, to discover the interactions between the G3Si PEG6000 dendrimer and plasma proteins. Human serum albumin (HSA) and Transferrin (Tf) are often selected as model proteins due to their critical roles in blood plasma and their relevance in the physiological processes affecting recognition by the immune system, cell internalization, toxicity, drug stability, half-life, biodistribution and BBB permeability^21,23,33–37^. This approach allowed direct comparisons with other nanovectors studied by different research teams and contributed to a broader and more standardized framework for evaluating nanoparticle behavior in biological systems. We extended our investigation to include interactions with plasma proteins to assess the studied dendrimer behavior in complex, physiologically relevant environments.

### Fluorescence quenching of HSA and Tf

**Fig. 2:**
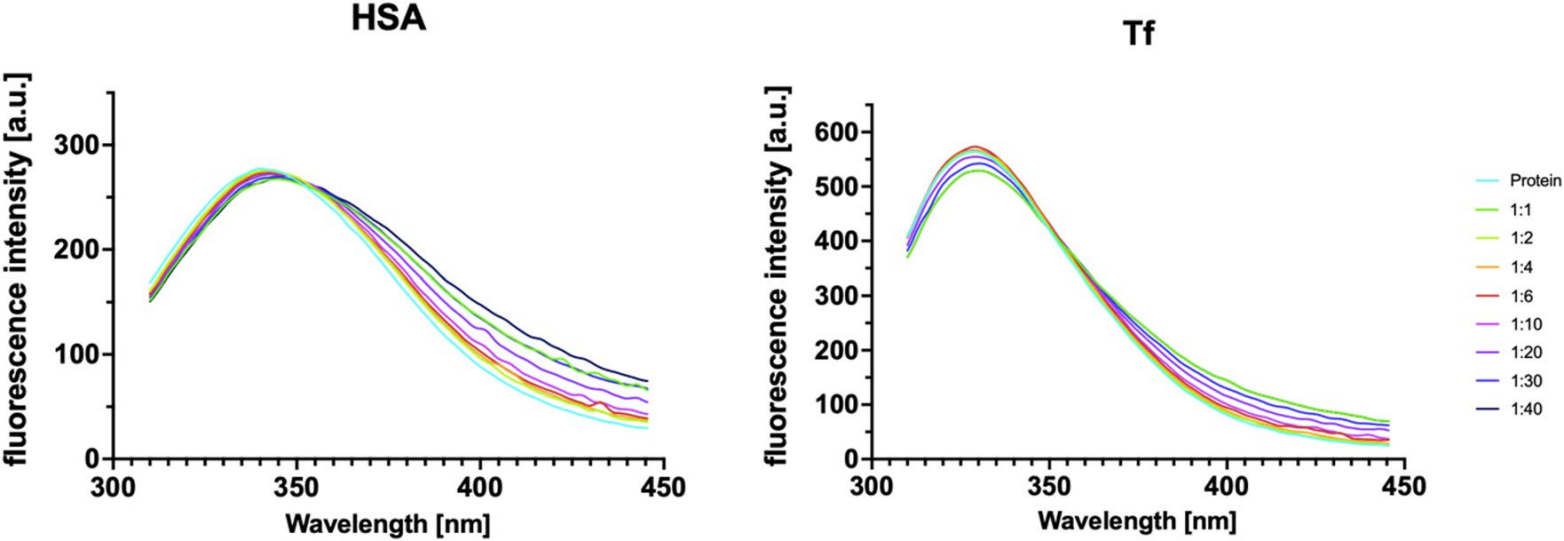
Fluorescence emission spectra of human serum albumin and transferrin in the presence of increasing concentrations of the G3Si PEG6000 dendrimer at a wavelength ranging from 310 to 445 nm.

We employed fluorescence quenching assay as it is a very sensitive method for providing a basis for quantitative measurements, giving insight into measuring drugs, drug carriers or xenobiotics, and protein interactions on a molecular level. The intrinsic fluorescence of proteins primarily arises from aromatic amino acid residues—tryptophan (Trp), tyrosine (Tyr), and phenylalanine (Phe). Changes in the emission spectrum of Trp resulting in fluorescence quenching often indicate alterations in protein conformation, excited state reactions, energy transfer, the formation of ground state complexes, and collision quenching^38^. From a structural perspective, HSA is composed of a single amino acid chain that folds into three homologous α-helical domains labeled I, II, and III. Each domain contains ten helices, which are further divided into two subdomains: a six-helix subdomain (A) and a four-helix subdomain (B). The first four helices in both subdomains form similar anti-parallel α-helix bundles. A single tryptophan residue is located in subdomain IIA. As anticipated, gradually adding the dendrimer to the HSA protein solution led to a decrease in tryptophan fluorescence intensity (Fig. 2). In the same manner, the red shift of the fluorescence spectra was observed.

Building upon our findings with HSA, our investigation was extended to explore the interactions between the dendrimer and Tf. This analysis allowed us to compare and contrast the dendrimer’s behavior with different protein targets, providing a more comprehensive understanding of its potential biological interactions. Tf is a bilobal protein, with each lobe composed of two domains consisting of a series of α-helical subdomains arranged around a central β-sheet scaffold. Tf contains eight tryptophan residues, three located in the N-lobe and five in the C-lobe^39^. A sequence of initial rapid quenching, recovery and gradual diminution of fluorescence was observed. These observations suggest a complex, multi-step interaction between the dendrimer and tested proteins, potentially involving multiple binding sites. The initial rapid quenching followed by fluorescence recovery may indicate conformational changes in the protein structure or rearrangement of the protein-dendrimer complex. The subsequent slow diminution in fluorescence intensity likely represents a more gradual process of dendrimer-protein association or further structural modifications. The persistent red shift in the fluorescence spectra corroborates the hypothesis of alterations in the local environment of the tryptophan residue, potentially due to dynamic changes in protein conformation or dendrimer binding.

Fluorescence quenching can be categorized into two distinct mechanisms: static and dynamic. Dynamic quenching (collisional quenching) arises from the collisions between these molecules when the fluorophore returns to its ground state without emitting a photon, thus decreasing fluorescence intensity. Static quenching occurs in the ground state when the quencher binds to the fluorophore, forming a non-fluorescent complex that effectively reduces the overall fluorescence. The type of quenching can be speculated based on the Stern–Volmer plots. The classical Stern-Volmer equation describes the decrease in fluorescence intensity as a function of the concentration of a collisional quencher:

$$\:\raisebox{1ex}{${F}_{0}$}\!\left/\:\!\raisebox{-1ex}{$F$}\right.=1+{k}_{q}{\tau\:}_{0}\left[Q\right]$$

In this equation, $\:{F}_{0}$ and $\:F$ represent the fluorescence intensities in the absence and presence of the quencher, respectively. The term $\:{k}_{q}$ denotes the bimolecular quenching constant, while $\:{\tau\:}_{0}$ is the lifetime of the fluorophore in the absence of a quencher. The concentration of the quencher is indicated by $\:\left[Q\right]$. The Stern-Volmer quenching constant, $\:{K}_{sv}\:$, is defined as the multiplication of $\:{k}_{q}$ and $\:{\tau\:}_{0}$. It has been widely reported that the maximum bimolecular quenching rate constant for various quenchers interacting with biopolymers is approximately 2.0 × 10¹⁰ M⁻¹ s⁻¹, serving as a reference threshold for diffusion-controlled quenching processes^40,41^. Given that in our experiment, the $\:{k}_{q}$ values for the quenching of both studied proteins by the G3Si PEG6000 dendrimer exceed 2.0 × 10¹⁰ M⁻¹ s⁻¹, it can be inferred that, within the tested concentration range, the observed fluorescence quenching predominantly occurs via a static mechanism. While dendrimer–HSA interactions are likely governed by a linear quenching process, dendrimer–Tf interactions involve an additional variable. Tf contains eight Trp residues, each having potentially distinct microenvironments and varying degrees of accessibility to the quencher, resulting in a more complex fluorescence quenching behavior. The Stern–Volmer plot for the Tf–G3Si PEG6000 deviates from linearity toward the x-axis up to 1:10 molar ratio, also decreasing the square of the correlation coefficient (R²). This non-linearity likely arises from conformational changes in the tertiary structure of Tf, leading to differential accessibility of fluorophores to the quencher^38^.

**Fig. 3:**
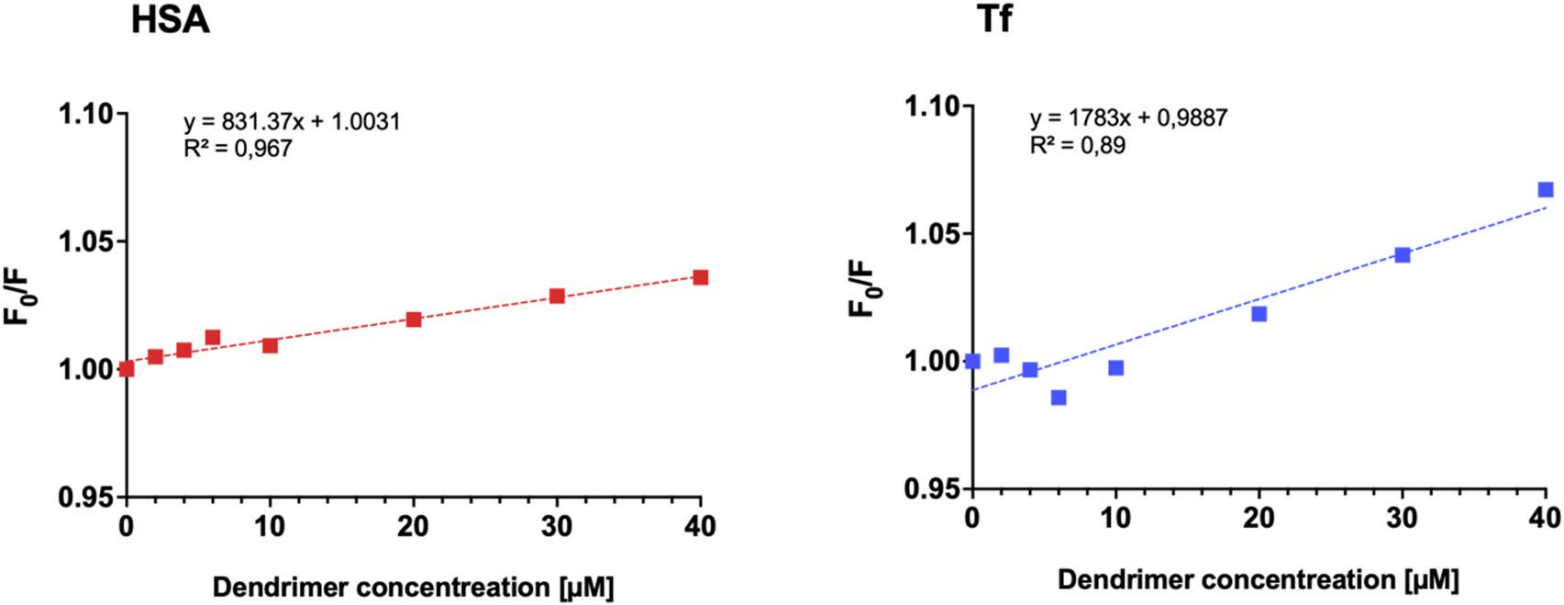
Stern–Volmer plots of the human serum albumin and transferrin fluorescence quenching in the presence of increasing concentrations of the G3Si PEG6000 dendrimer.

To determine the binding constant $\:{K}_{b}$, the experimental data were fitted to the following equation:

$$\:\text{l}\text{o}\text{g}\left(\raisebox{1ex}{${F}_{0}$}\!\left/\:\!\raisebox{-1ex}{$F$}\right.\right)=log{K}_{b}+nlog\left[Q\right]$$

where $\:n$ is the number of binding sites. As presented in Table 1, the $\:{K}_{sv}$ for the Tf-G3Si PEG6000 conjugate is approximately twofold higher than that for the HSA: G3Si PEG6000 conjugate, yielding a markedly steeper Stern–Volmer plot (Fig. 3). Consequently, this indicates that energy transfer occurs substantially more efficiently between Tf and the G3Si PEG6000 dendrimer compared to the interaction with HSA. Furthermore, the $\:{K}_{b}$ for the Tf-G3Si PEG6000 is nearly three times greater than that for the HSA conjugate, which suggests a markedly higher binding affinity between Tf and the G3Si PEG6000 dendrimer (Table 1). A comparative analysis of the $\:{k}_{q}$ and the $\:{K}_{sv}\:$for the tested dendrimer-HSA interaction reveals distinctive characteristics relative to other compounds documented in the literature. The $\:{k}_{q}$ value of β-Carboline silver compounds, which exhibit potential for crossing the BBB, indicates the quenching mechanism to be static with the $\:{k}_{q}$ value reaching up to 5.37 × 10^13^ M^–1^ s^–1^^42^. Similarly, studies on α-Cembrenediol, known for its neuroprotective effects, show the $\:{k}_{q}$ value reaching up to 1.49 × 10^11^ M^–1^ s^–1^, indicative of the static quenching mechanism of HSA^43^. Another well-characterized group of compounds includes the first-generation polyphenolic carbosilane dendrimers, which exhibit quenching rate constants of up to 1.27 × 10^11^ M^–1^ s^–1^, also suggesting quenching the HSA fluorescence via the static mechanism^44^. A study of tyrosine-modified polyethyleneimines designed for siRNA delivery exhibited $\:{k}_{q}$ values in the range from 4.6 × 10^12^ to 1.5 × 10^14^ M^–1^ s^–1^ for the different polymers studied, indicating also the static quenching mechanism of HSA^45^. These findings highlight how different physicochemical properties influence protein interactions. Compounds with similar structures or applications, such as polyphenolic carbosilane dendrimers, β-carboline silver complexes, and tyrosine-modified polyethyleneimines, exhibit strong and potentially biologically relevant interactions with serum albumins. Like the studied G3Si PEG6000 dendrimer, these compounds demonstrate high binding affinities and predominantly static quenching mechanisms, suggesting their potential to influence protein conformation and function in biological environments.

**Table 1:** Protein-dendrimer interaction parameters were determined from fluorescence quenching data.

Protein	$\:{\varvec{K}}_{\varvec{s}\varvec{v}}\:\left[{\varvec{M}}^{-1}\right]$	$\:{\varvec{K}}_{\varvec{q}}\:\left[{\varvec{M}}^{-1}{\varvec{s}}^{-1}\right]$	$\:{\varvec{K}}_{\varvec{b}}\:\left[{\varvec{M}}^{-1}\right]$
HSA	$\:8.3 \times {10}^{2}$	$\:1.5 \times {10}^{11}$	$\:3.3 \times {10}^{2}$
Tf	$\:1.8 \times {10}^{3}$	$\:5.9 \times {10}^{11}$	$\:9.6 \times {10}^{2}$

### Circular dichroism of HSA and Tf

Circular dichroism (CD) is a spectroscopic technique based on the differential absorption of left- and right-handed circularly polarized light by chiral molecules. CD measurements of proteins affected by nanovectors in the far-ultraviolet regions are primarily influenced by the n→π* and π→π* electrons energy transition of their amide groups. These transitions are sensitive to the geometry changes of the polypeptide backbone, resulting in spectral changes that provide insight into alterations in the secondary structure.

**Fig. 4:**
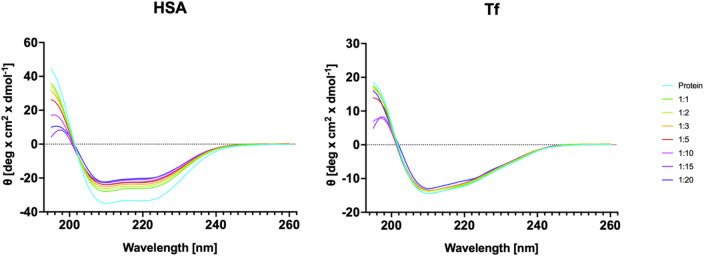
Circular Dichroism spectra of human serum albumin and transferrin following the addition of dendrimer G3Si PEG6000 at a wavelength ranging from 195 to 260 nm.

**Fig. 5:**
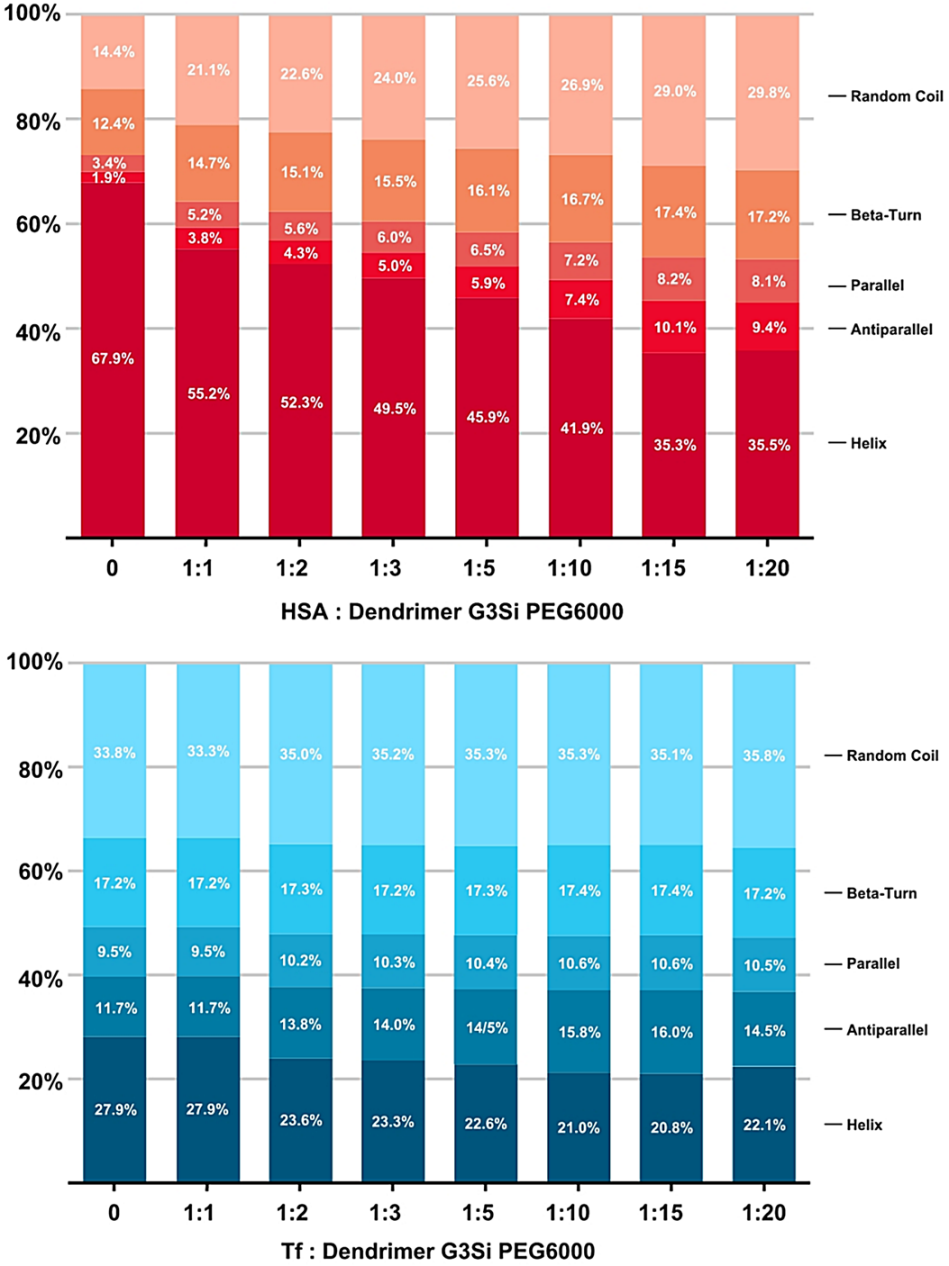
Alterations in the secondary structure of human serum albumin (red) and transferrin (blue) upon interaction with dendrimer G3Si PEG6000. The analysis highlights changes in specific structural elements, such as α-helices, β-sheets, parallel, anti-parallel arrangement, and random coils, potentially indicating conformational rearrangements induced by dendrimer binding.

CD fluorescence spectra revealed that both HSA and Tf undergo conformational changes upon interaction with the G3Si PEG6000 dendrimer (Fig. 4). The CD spectra of HSA in the absence of the studied dendrimer due to the π→π* transition show a single positive ellipticity at 193 nm and a negative band at 208 nm. The second negative band at 222 nm is due to the n→π* characteristic for the α-helix rich protein. The observed spectra flattening indicative of loss of α-helical content were most pronounced after the initial addition of the dendrimer in the 1:1 ratio of HSA to dendrimer and continued to evolve, with a less pronounce shift occurring at the 1:15 ratio of HSA to dendrimer. This pattern was further confirmed by deconvolution analysis, which provided estimates of the secondary structure composition for each CD spectrum (Fig. 5). The α-helical content of HSA decreased by approximately 13% at a 1:1 HSA-to-dendrimer ratio, with a further reduction of about 6.2% at the 1:15 ratios of HSA to dendrimer. The structural changes in HSA were more pronounced than those observed for Tf. The Tf spectra in the absence of the studied dendrimer due to the n→π* transition show a single positive ellipticity at 195 nm and a negative band at 215 nm, indicating protein with a high β-sheet secondary structure content. The first significant change in the Tf structure occurred at a 1:2 Tf- dendrimer ratio, leading to a 3.7% reduction in α-helical content. A second major structural shift was observed at a 1:20 ratio, where a slight increase in α-helical content was accompanied by a decrease in anti-parallel content.

The findings suggest a multi-step interaction mechanism for the formation of the G3Si PEG6000–protein conjugate. A multi-step conformation shift typically suggests the presence of multiple domains affected. We hypothesize that initially, the dendrimer associates with the protein, partially unfolding it, and upon the increase of the dendrimer concentration, the conjugate transitions into a highly misfolded state. This integrated perspective allows for further comparisons across different nanovectors and research studies, advancing the understanding of nanoparticle behavior and protein interaction mechanisms. The conjugation of the protein with the polyphenolic carbosilane dendrimers at 1:10 molar ratio reduces the α-helical content up to 53.40%^44^. The effect of the β-Carboline silver compounds on the HSA structure shown similar conformational shift reaching 35.63% of HSA α-helical structure at the molar ratio of 1:5000 HSA to tested compounds^42^. Similarly, the dendrimer G3Si PEG6000, pegylated gold nanoparticles intended for nucleic acid delivery across the BBB markedly reduce the α-helical content of HSA, while Tf shows only minor changes in secondary structure^46^.

### Isothermal calorimetric titration of HSA

**Fig. 6:**
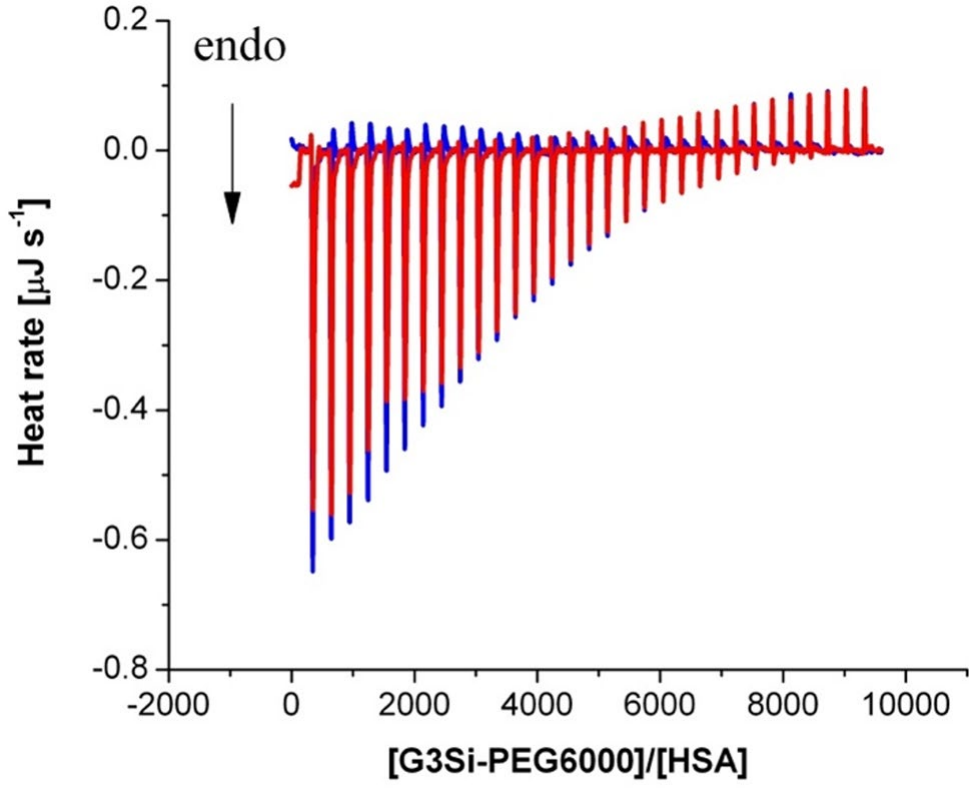
Curves of thermal power as a function of time during titration of 10 µM HSA solution with 0.2 mM G3Si PEG6000 dendrimer solution (red peaks) and dilution of 0.2 mM dendrimer solution into buffer without HSA (blue peaks). All titrations were carried out at 25 °C in aqueous 10 mM phosphorous buffer solution pH 7.4. Downward peaks describe endothermic heat effects.

**Fig. 7:**
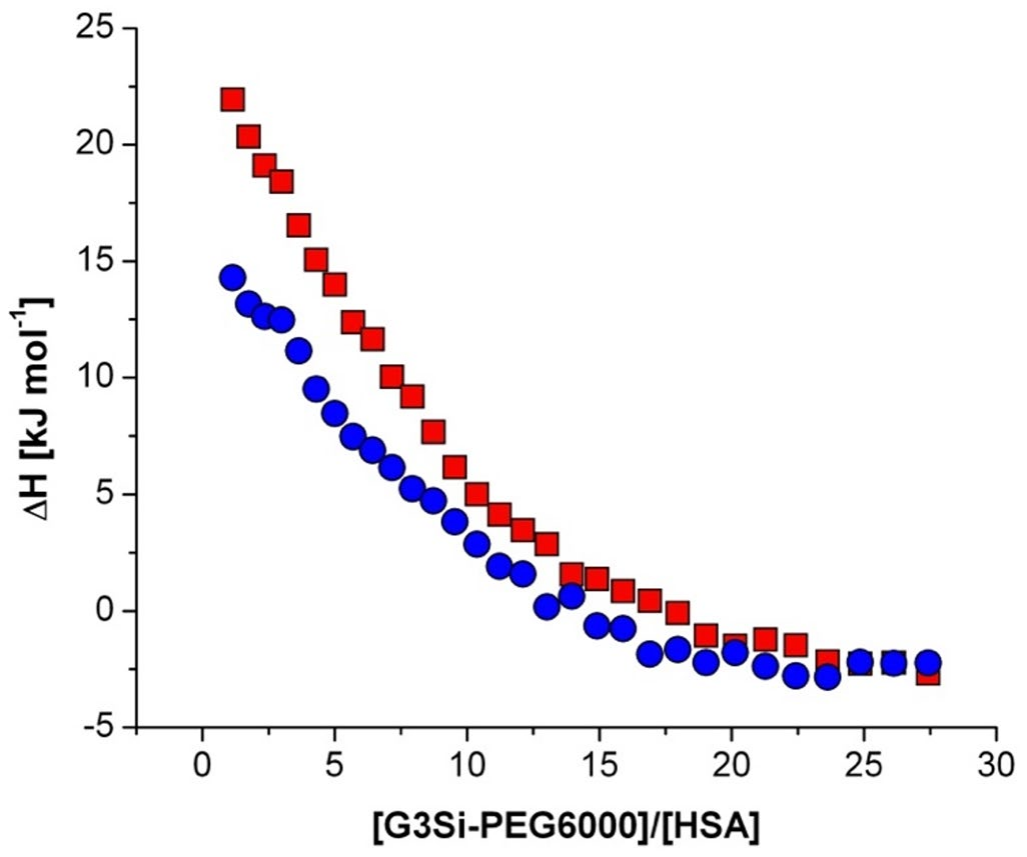
Integrated thermal effects of the ITC titration of 10 µM HSA solution with 0.2 mM G3Si PEG6000 dendrimer solution (red squares) and corresponding effects of dilution of dendrimer (blue circles) in aqueous 10 mM phosphorous buffer solution pH = 7.4 at 25 °C as a function of the dendrimer to HSA mole ratio.

**Fig. 8:**
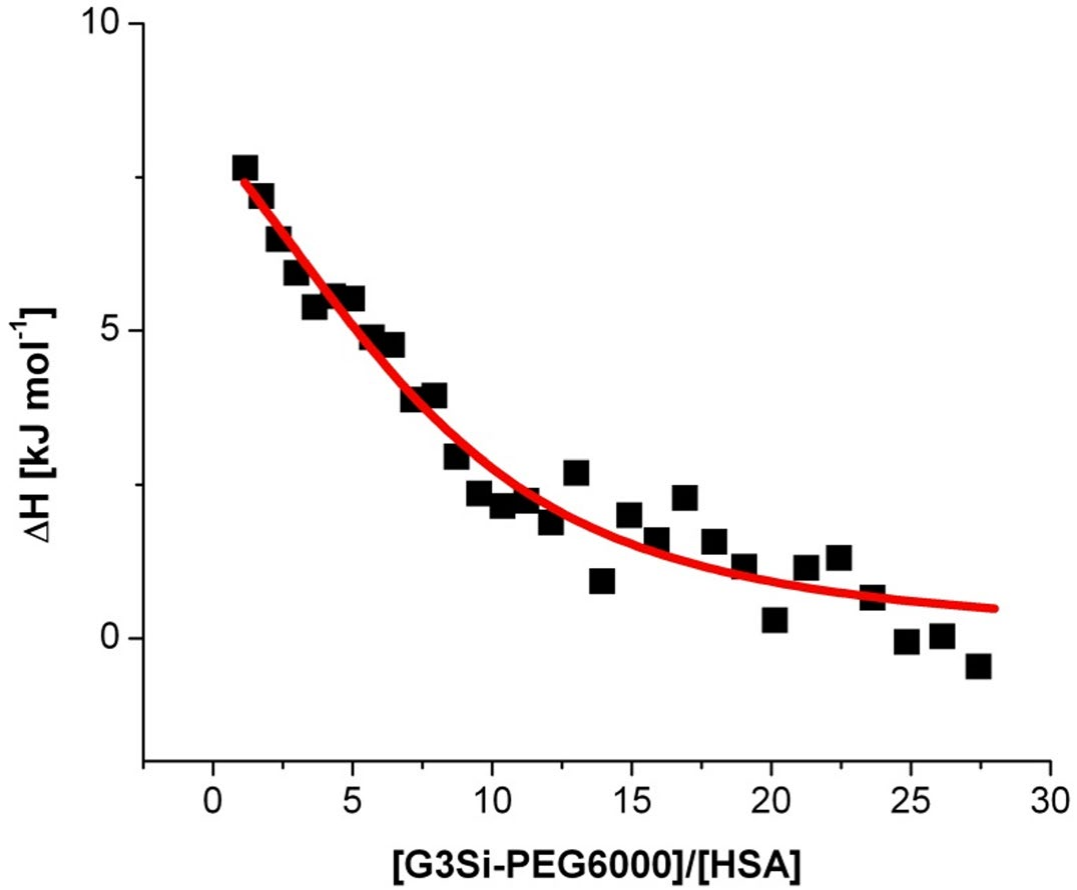
Thermal effects of direct interactions (per mole of injectant) between HSA and G3Si PEG6000 dendrimer as a function of the dendrimer to HSA mole ratio in aqueous 10 mM phosphorous buffer solution pH 7.4 at 25 °C the ITC curve of HSA binding interaction with studied dendrimer was described with the identical-site model (solid lines).

To determine the reaction thermodynamics and kinetics of protein–dendrimer interactions, isothermal titration calorimetry (ITC) was employed. In this and consecutive experiments, HSA was chosen as a complexation partner as its interactions with the studied dendrimer are strong and profoundly affect protein secondary structure compared to dendrimer-Tf interactions. After injections of the G3Si PEG6000 dendrimer solution into a calorimetric cell containing the HSA solution, the heat is measured by determining the power required to maintain a constant temperature with respect to a reference solution of dendrimer titrated into a buffer. The reaction heats tend to zero as the titration progresses, and the binding sites become saturated. The heat rate as a function of time is presented in Fig. 6. The heat rates of both titrations were integrated into thermal effects (Fig. 7). The enthalpy of the HSA interaction with the studied dendrimer (Fig. 8), corrected by the dendrimer dilution effects, were analyzed in the NanoAnalyze software by non-linear multiparameter regression using the identical-site model. The stoichiometric parameters, the binding constant, and thermodynamic functions are summarized in Table 2. The stoichiometric parameter indicates a multivalent interaction where each HSA molecule can bind 9 ± 3 dendrimer molecules. This is in reasonable agreement with the CD spectral shifts, which indicate that a single HSA molecule undergoes a major secondary structure conformation change, at least at the 1:15 HSA: dendrimer molar ratio. The determined binding constant (logK = 4.47 > 3) reflects the high affinity between the dendrimer and each HSA active site. Thermodynamically, the process is driven by a highly favorable entropy change ($\:T\varDelta\:\:S$ = 36 kJ mol^–1^), which indicates a significant disorder introduced into the system, likely due to several contributions comprising the changes of protein structure caused by its interactions with dendrimer and the changes in dendrimer macromolecule structure including the changes in PEG6000 substituent itself as well as the release of water molecules or ions during dendrimer binding with the HSA active sites. This positive entropy change overcomes the unfavorable positive enthalpy of binding ($\:\varDelta\:H$ = 11 kJ mol^–1^), which suggests that the direct interactions between the HSA and dendrimer are weakly endothermic, likely due to desolvation or conformational adjustments. The overall Gibbs free energy change $\:(\varDelta\:G$ = − 26 kJ mol^–1^) confirms that the binding is thermodynamically spontaneous, with entropy as the driving force.

**Table 2:** Calorimetrically determined stoichiometric parameter n, binding constant K (M^–1^), and standard thermodynamic functions of HSA interaction with G3Si PEG6000dendrimer in 10 mM phosphorous buffer solution pH 7.4 at 25 °C.

Dendrimer	$\:\varvec{n}$	$\:\varvec{l}\varvec{o}\varvec{g}\varvec{K}$	$\:\varDelta\:\varvec{H}$ (kJ mol^–1^)	$\:\varvec{T}\varDelta\:\varvec{S}$ (kJ mol^–1^)	$\:\varDelta\:\varvec{G}$ (kJ mol^–1^)
G3Si PEG6000	9 ± 3	4.47	11	36	–26

### Atomic force microscopy of HSA and plasma

**Fig. 9:**
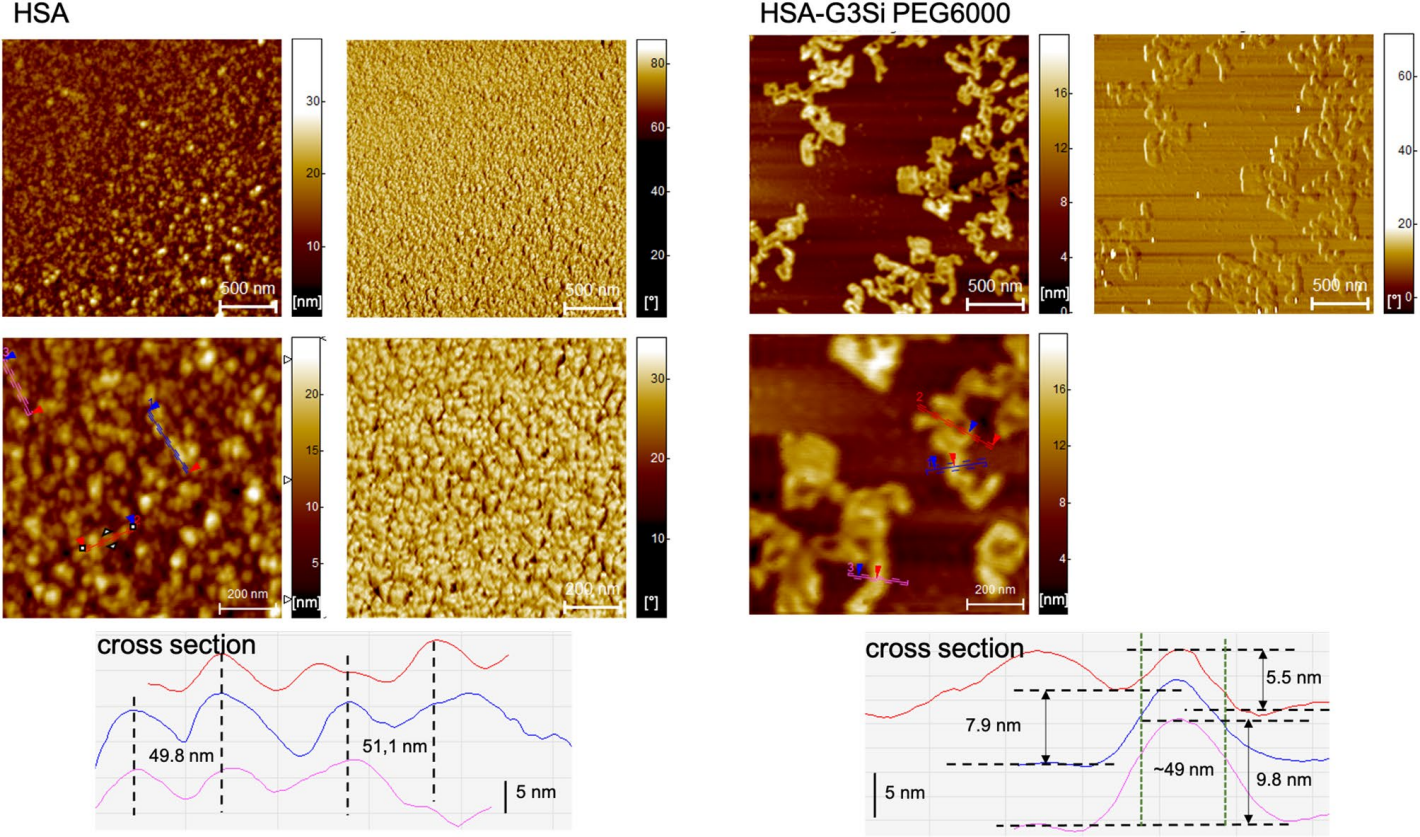
AFM height and phase images of HSA (left panel) and its conjugates with the dendrimer G3Si PEG6000 (right panel). Corresponding cross-section line profiles (taken along the colored guide lines) are shown beneath each image.

Atomic force microscopy (AFM) imaging was employed to investigate the morphology of HSA-dendrimer conjugates. HSA, in the absence of dendrimer conjugation, exhibits a monodisperse, uniform globular morphology indicative of its intrinsic structural stability (Fig. 9). The mica substrate was essentially not visible, indicating near-continuous coverage by HSA. This made it impossible to determine absolute feature height (no exposed baseline); only lateral dimensions were gauged from cross-sections. Consequently, the ~ 50 nm “diameter” observed for HSA alone should be interpreted as clusters of albumins on tightly packed monolayers inflated by the tip-sample convolution rather than single proteins. Upon interaction with the G3Si PEG6000 dendrimer, significant structural alterations are observed, marked by the emergence of elongated aggregates. This suggests that the dendrimer disrupts the native state of HSA, facilitating protein aggregation and secondary and tertiary structural rearrangements. Globular proteins, including HSA, can undergo conformational changes upon external perturbation, which leads to partial unfolding and subsequent aggregation into fibrillar structures^47^. The conformational rearrangement of HSA, affected by interactions with G3Si PEG6000, appear as linear or curvilinear assemblies of multiple globules and resembles semiflexible albumin fibrils observed in multiple studies^48,49^. The dendrimer-induced aggregates exhibit lateral widths on the order of ~ 50 nm (similar to the HSA-alone). Individual aggregates were analyzed, yielding heights ranging from approximately 5.5 nm up to 9.8 nm. We emphasize that the height values are much more accurate indicators of aggregate thickness than lateral size, since the vertical measurement is less prone to tip broadening or edge convolution. Nonetheless, the appearance of ~ 8 nm high, ~ 50 nm wide, elongated clusters in the HSA conjugated with dendrimer sample is a robust, reproducible finding. Dendrimer addition clearly produces a new morphology: linear aggregates that were absent in HSA alone. The observed aggregates likely result from intermolecular interactions that promote β-sheet formation, a hallmark of protein misfolding and aggregation. These changes align with findings from the CD spectroscopy, which also reveals rearrangement in its secondary structure.

**Fig. 10:**
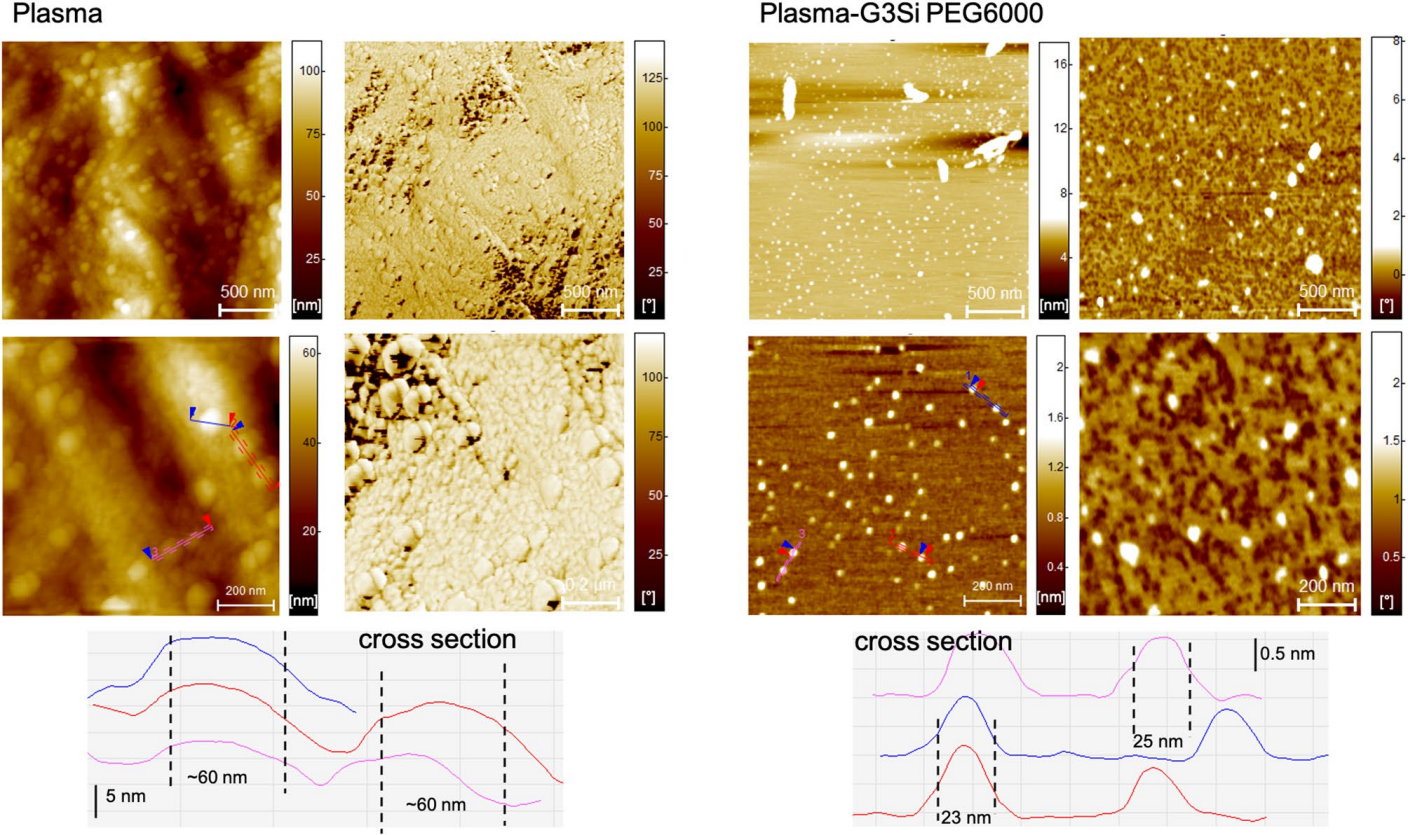
AFM height and phase images of plasma (left panel) and its conjugates with the dendrimer G3Si PEG6000 (right panel). Corresponding cross-section line profiles (taken along the colored guide lines) are shown beneath each image.

Nanovectors *in vivo* encounter a protein-rich environment where their interactions can have dual effects on protein aggregation. On the one hand, the dendrimers may act as scaffolds or catalysts, locally concentrating proteins and enhancing aggregation processes such as fibril nucleation and elongation. On the other hand, when the protein concentration is high, the dendrimer surface becomes saturated with proteins that competitively bind to it. This competitive binding can block the active sites necessary for further protein-protein interactions, thereby inhibiting fibril formation^50^. Consequently, dendrimers’ net effect *in vivo* depends on the balance between their ability to concentrate proteins versus the potential to sequester and stabilize them, which can influence both the extent and the nature of aggregation observed in biological systems. It is also important to denote that a multitude of factors like blood flow, axial margination, protein corona composition, etc., could potentially alter the interactions.

Utilizing an *in vitro* approach, the AFM phase images reveal multiple areas with notable morphological changes, where the plasma structure transitions from predominantly globular forms to more elongated aggregate structures similar to the changes observed upon the addition of dendrimer G3Si PEG6000 to HSA (Fig. 10). Upon close inspection, the elongated aggregate arrangements are composed of smaller, rod-like units. Each globular node along these chains had an apparent lateral size of ~ 23 nm on average, significantly smaller than the ~ 60 nm globules seen in plasma without introduction of the studied dendrimer. This suggests that the dendrimer may reorganize the native plasma protein into finer units. This change suggests that by altering protein secondary and tertiary structures, the dendrimer may start a nucleation process by locally increasing protein concentration or inducing conformational changes that favor protein misfolding, oligomerization, and protofibril formation. Height images demonstrate an enhanced regularity of plasma dispersion across the entire surface of the sample compared to the more irregular protein distribution prior to the dendrimer addition. This improved dispersion could indicate the stabilization or reorganization of plasma components facilitated by interactions with the dendrimer. The improved dispersion implies that the dendrimer might simultaneously limit uncontrolled aggregate growth, potentially by reorganization of plasma components.

### Transmission electron microscopy of HSA and plasma

**Fig. 11:**
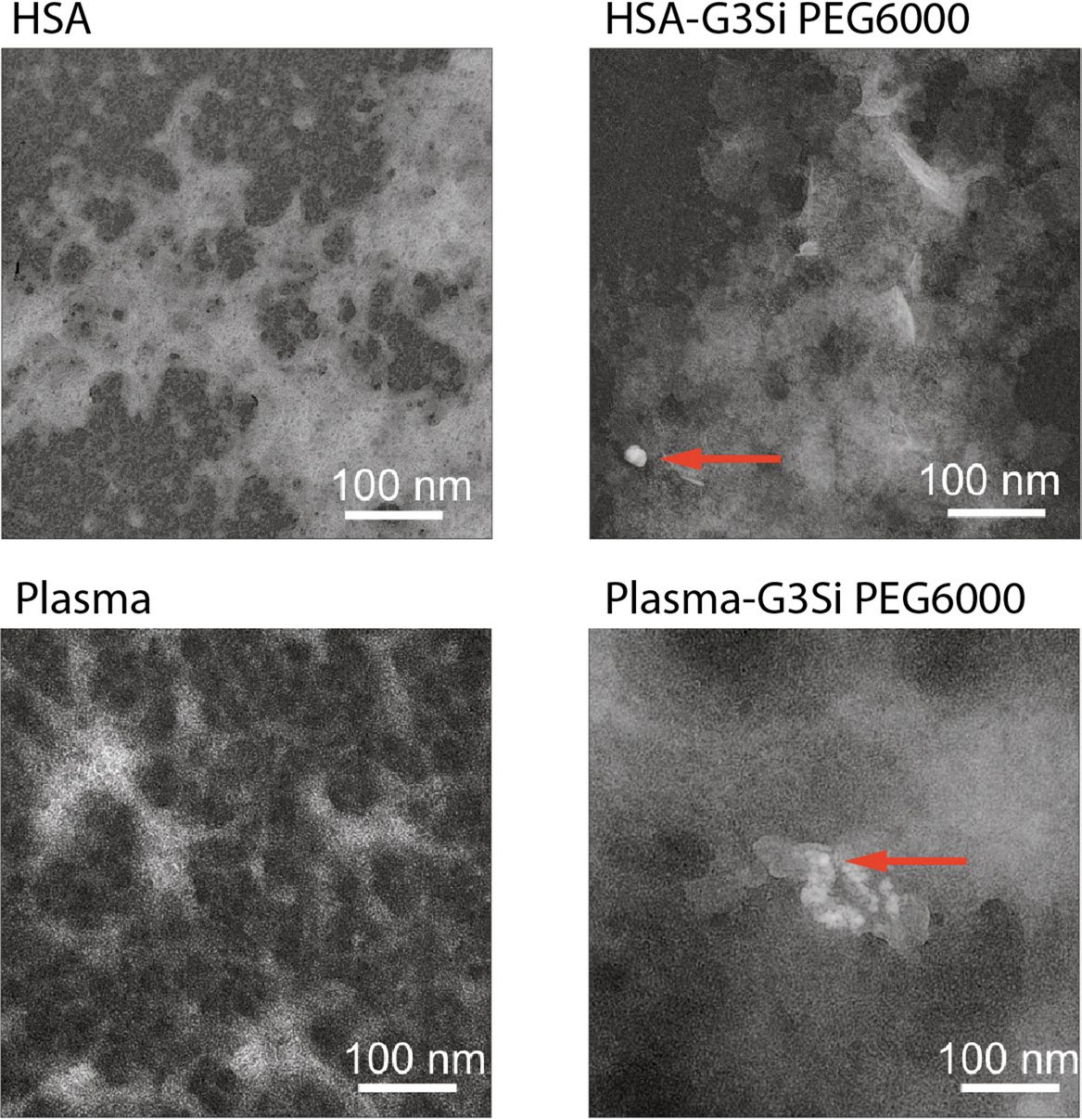
Transmission electron microscope images HSA (top left), HSA in the presence of the dendrimer G3Si PEG6000 in 9:1 molar ratio (top right), plasma (bottom left), and the G3Si PEG6000 dendrimer in plasma environment (bottom right). A magnification of 100 000 × was used to analyze the samples. Arrows point at partial aggregation into denser protein assemblies. TEM micrographs of the not conjugated dendrimer G3Si PEG6000 are available in our prior work^32^.

Transmission electron microscopy (TEM) provided complementary insights into the structural changes induced by the interaction of dendrimer G3Si PEG6000 with HSA and plasma (Fig. 11). Free albumin exhibited an amorphous aggregate morphology. The differences in HSA images observed in AFM and TEM can be attributed to the distinct principles and preparation requirements of these techniques. As a morphological reference, representative TEM micrographs of the not conjugated dendrimer G3Si PEG6000 acquired under the same negative-stain conditions are available in our prior work^32^. Upon dendrimer addition, the structures became slightly more compact, suggesting that the dendrimer interacts with HSA, which induces partial aggregation into denser protein assemblies. In plasma, which inherently exhibited more complex and intertwined structures compared to HSA alone, the introduction of the dendrimer led to a more dispersed protein arrangement, resembling the patterns observed in AFM images, with localized interactions between the dendrimer and plasma proteins visible. AFM and TEM morphology report cannot, on their own, confirm protein fibrillation; orthogonal assays are required for that determination.

## Conclusions

A key reason for the longstanding interest in nanovector–protein interactions is the pursuit of effective and safe therapeutic delivery. Unraveling the underlying mechanisms is fundamental to achieving controlled biodistribution and fully realizing the potential of nanomedicine. Our studies reveal that G3Si PEG6000 dendrimers interact with model proteins in distinct ways. Fluorescence quenching data indicate that the dendrimer binds Tf with higher affinity than HSA, suggesting that dendrimer binding induces more pronounced tertiary conformational changes in Tf. In contrast, circular dichroism analyses reveal that the G3Si PEG6000 dendrimer binding with the model proteins induces a more pronounced effect on the HSA secondary structure. The interactions of the dendrimer with both of the proteins show distinct, multi-step conformational modifications of these proteins, implicating the involvement of multiple binding domains. Isothermal titration calorimetry studies also confirm that the HSA–G3Si PEG6000 dendrimer interaction is characterized by multivalent binding and is driven by a favorable entropy change. Together with AFM and TEM imaging, these findings suggest that at high concentrations, the G3Si PEG6000 dendrimer can alter the protein’s secondary and tertiary structures, potentially reorganizing plasma components and initiating nucleation processes that render the proteins more susceptible to misfolding. Though inherently complex, the interaction profile of G3Si PEG6000 dendrimers mirrors key characteristics observed in referenced and well-established nanocarrier systems. The study highlights the intrinsic and multifaceted nature of protein–nanovector interactions and reinforces the suitability of this platform for continued investigation in drug delivery and precision medicine. A multitude of physiological processes, including blood flow, corona remodeling through competitive adsorption, and systemic clearance pathways, are likely to influence these interactions. Therefore, extrapolating from these *in vitro* results to *in vivo* behavior should be done with caution. These dendrimer-induced, concentration-dependent alterations must be further assessed within a broader biological context, where the vast and dynamic network of molecular interactions in the circulatory system dictates a spectrum of potential outcomes. Future studies of the G3Si PEG6000 dendrimer will extend these insights by including morphotic elements to further describe the impact of dendrimer–protein interactions on the dendrimer’s therapeutic delivery and safety.

## Data Availability

The datasets generated during and/or analysed during the current study are available from the corresponding authors on reasonable request. Correspondence and should be addressed to S.Z or K.M.

## Abbreviations

AFM: Atomic force microscopy
APOE4: Apolipoprotein E4
BBB: Blood-brain barrier
CD: Circular dichroism
CNS: Central nervous system
HSA: Human serum albumin
ITC: Isothermal calorimetric titration
MPS: Mononuclear phagocyte system
PEG: Polyethylene glycol
Phe: Phenylalanine TEM
Trp: Tryptophan
Tf: Human transferrin
Tyr: Tyrosine
